# TROY interacts with RKIP to promote glioma development

**DOI:** 10.1038/s41388-018-0503-x

**Published:** 2018-10-18

**Authors:** Xiujie Liu, Yinghui Bao, Wei Meng, Ping Yang, Yi An, Jie Ma, Yujie Tang, Zhigang Liu, Yan Lu, Jianfeng Zhou, Yong Zhang, Jifeng Feng, Xiaofei Gao, Zhida Su, Yingyan Pu, Cheng He

**Affiliations:** 1 Institute of Neuroscience, Key Laboratory of Molecular Neurobiology of Ministry of Education and the Collaborative Innovation Center for Brain Science, Second Military Medical University, Shanghai, 200433 China; 2 Department of Neurosurgery, Ren Ji Hospital, Shanghai Jiao Tong University, School of Medicine, Shanghai, 200127 China; 3 Department of Pediatric Neurosurgery, Xin Hua Hospital Affiliated to Shanghai Jiao Tong University, School of Medicine, Shanghai, 200092 P.R. China; 4 Instrumental Analysis Center, School of Pharmacy, Fudan University, Shanghai, 201203 China; 5 Department of Therapeutic Oncology, Yale University School of Medicine, New Haven, CT USA; 6 Key Laboratory of Cell Differentiation and Apoptosis of National Ministry of Education, Department of Pathophysiology, Shanghai Jiao Tong University, School of Medicine, 280 South Chongqing Road, Shanghai, 200025 P.R. China; 7 Department of Radiation Oncology; Phase 1 Clinical Trial Ward, The Fifth Affiliated Hospital, Sun Yat-sen University, Zhuhai, Guangdong Province, 519001 China

**Keywords:** Cancer, Cell growth

## Abstract

TROY is a component of the Nogo receptor complex and plays the key role in neuronal survival, migration, and differentiation. Here, we show the up-regulation of TROY in human glioma tissues and cells. Inhibition of TROY expression slowed glioma development in vivo and in vitro. Raf kinase inhibitor (RKIP) was found to interact with TROY. The physical interaction of TROY/RKIP was confirmed via co-immunoprecipitation (co-IP) assays. Furthermore, we found that the TROY/RKIP interaction was enhanced by fetal bovine serum (FBS) exposure, and TROY knockdown also led to down-regulation of NF-κB. Finally, disruption of the TROY/RKIP interaction using the TAT-TROY (234–371 aa) protein reduced the glioma development in xenografted mice. This suggests the TROY/RKIP interaction is a potential target for therapy of gliomas.

## Introduction

The yearly worldwide incidence of primary brain tumors leads to about 7% of premature life span lost prior to the age of 70 [1–4]. Gliomas are the most common among brain tumors. Gliomas are graded as I to IV according to atypia, mitoses, necrosis, and microvascular enrichment. Grade I-II can have much longer time to progression than grade III-IV. High-grade gliomas are more aggressive and malignant, characterized as earlier infiltration, more resistance to therapy, and poorer outcomes [5, 6]. Though the incidence of glioma continue to rise, therapies for gliomas still largely consist of surgery, radiotherapy, and chemotherapy with limited advancements in recent decades [7]. Novel therapeutic approaches to gliomas are sorely needed. Uncontrolled cellular proliferation is a hallmark of glioma [8, 9]. Though, the mechanism of the abnormal proliferation in glioma cells remains largely unknown.

TROY, or tumor necrosis factor receptor 19 (TNFRSF19), is an orphan receptor of the TNFR superfamily [10, 11]. TROY has been widely reported as a co-receptor which activates the RhoA and inhibits neurite outgrowth [12, 13]. TROY-knockout mice show the significant reduced neurite outgrowth inhibition in the presences of myelin-associated inhibitory factors [14, 15]. Additionally, expressoin of TROY has been implicated in various developmental systems, including hair follicle cell development [16], tooth development [17], melanoma cell growth [18], and inflammation [19]. TROY expression appears to be restricted to certain brain regions in adults [10]. Recently, several genome-wide association studies identified that increased expression of TROY was associated with susceptibility to nasopharyngeal carcinoma and lung malignancies [20, 21], suggesting a role of TROY in tumorigenesis.

In this report, the effect of TROY on glioma development was investigated. The up-regulation of TROY expression was observed in human glioma tissues. TROY was found to interact with RKIP to promote glioma development by enhancing downstream NF-κB signaling. Moreover, the disruption of TROY/RKIP interaction reduced the growth of xenografted glioma in nude mice.

## Results

### TROY Expression is Upregulated in Human Glioma

**Fig. 1:**
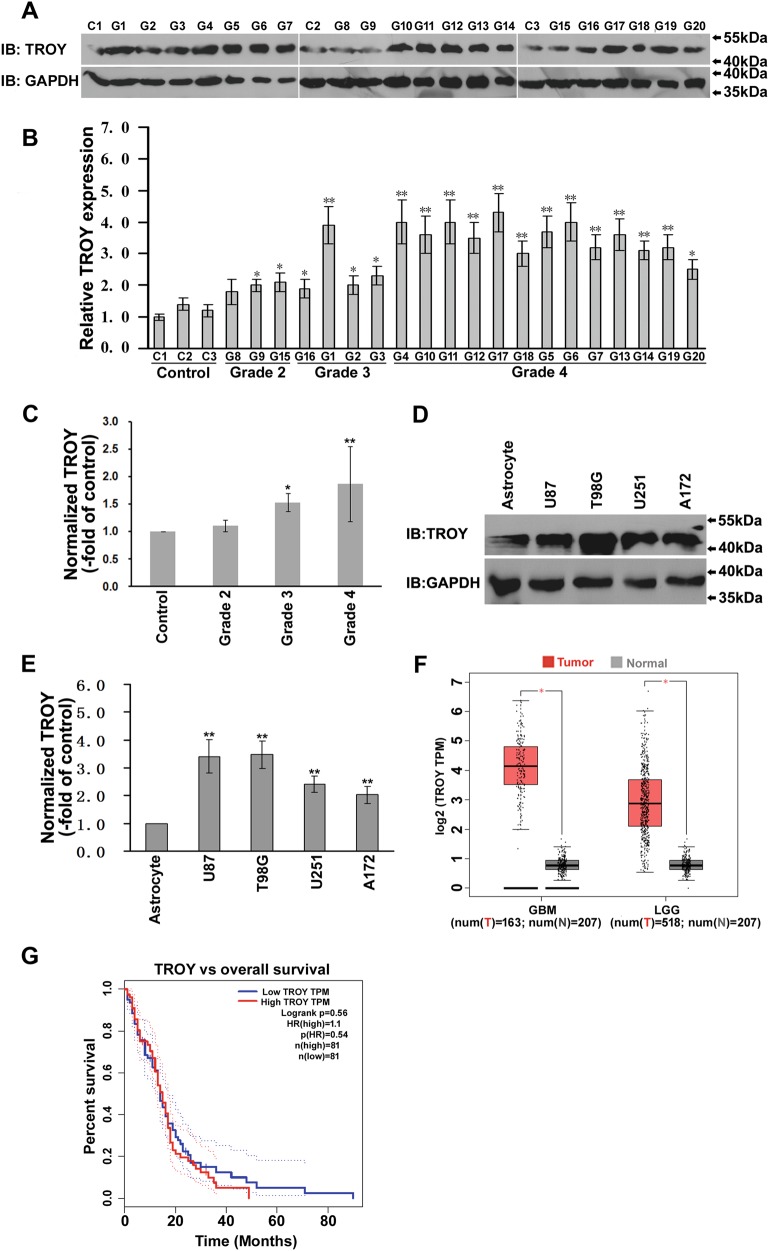
Expression of TROY protein in human glioma cells and tissues. (**a**) TROY levels in normal tissues (C1–3) and glioma (Grade2, G8,9,15; Grade 3, G1–3 and 16; Grade 4, G4–7, 10–14 and 17–20). GAPDH was an internal control. (**b**) RT-PCR of TROY in normal and glioma tissues. Relative TROY expression are normalized to that of the C1 sample. Three different portions of samples were examined. (**c**) The band intensities of *a* are quantified followed by normalized to the control. (**d**) Western blot of TROY expression in U87, T98G, U251 and A172 cell lines. Normal human astrocyte was the control. (**e**) The band intensities of d are quantified and normalized to the control. (**f**) The expression of TROY in human glioblastoma multiforme or brain lower grade glioma compared with normal tissues in TCGA database. The database were analyzed by GEPIA (http://gepia.cancer-pku.cn/) according to the previous report [24]. (**g**) The Kaplan–Meier analysis of survival in GBM in TCGA database

We initially found TROY to be widely expressed in neuroglia, consistent with previous reports [22, 23]. Total proteins were extracted from 20 primary glioma samples and 3 normal brain tissue samples. TROY expression were increased in high grade *vs* normal tissues by Western Blot (Fig. 1a, c) and quantitative RT-PCR (Fig. 1b). We also detected protein expression of TROY in 4 different glioma cell lines. TROY expression were higher than in normal astrocytes (U87, P < 0.001; T98G, P < 0.001; U251, P = 0.003; A172, P = 0.006) (Fig. 1d, e). The data suggest TROY expression are upregulated in the glioma cells. Additionally, We examined the correlation between levels of TROY and patient survival among different grades of glial tumor in the patient specimens from public database including The Cancer Genome Atlas (TCGA) and The Genotype-Tissue Expression (GTEx) data. Gene Expression Profiling Interactive Analysis revealed TROY was significantly overexpressed in human glioblastoma multiforme or brain lower grade glioma tissues compared to normal glial tissues (Fig. 1f). Additionally, high TROY expressions was associated with worse survival (Fig. 1g).

### Knock-down of TROY Suppresses the Growth of Glioma Cells

To explore the biological functions of TROY, we prepared three shRNAs (shRNA1–3#) that specifically target human TROY. U87 cells stably expressing TROY shRNAs were constructed. The TROY protein level in U87 cells was significantly reduced by shRNA 2# (P < 0.001) and shRNA3# (P < 0.001) (Supplementary Fig. S1a, b). In colony formation assays, transfection with TROY shRNA2# and 3# significantly inhibited the proliferation of U87 cells, which were reflected through the number and size of colonies after crystal violet staining (Supplementary Fig. S1c). The growth of colonies were quantified by measuring OD595, which show that shRNA2# and 3# transfection substantially decreased the OD595 values of the colonies in the U87 cells (for shRNA2#, P = 0.04; for shRNA3#, P = 0.02) (Supplementary Fig. S1d). These results suggest the specific knock-down of TROY suppresses glioma cell growth.

**Fig. 2:**
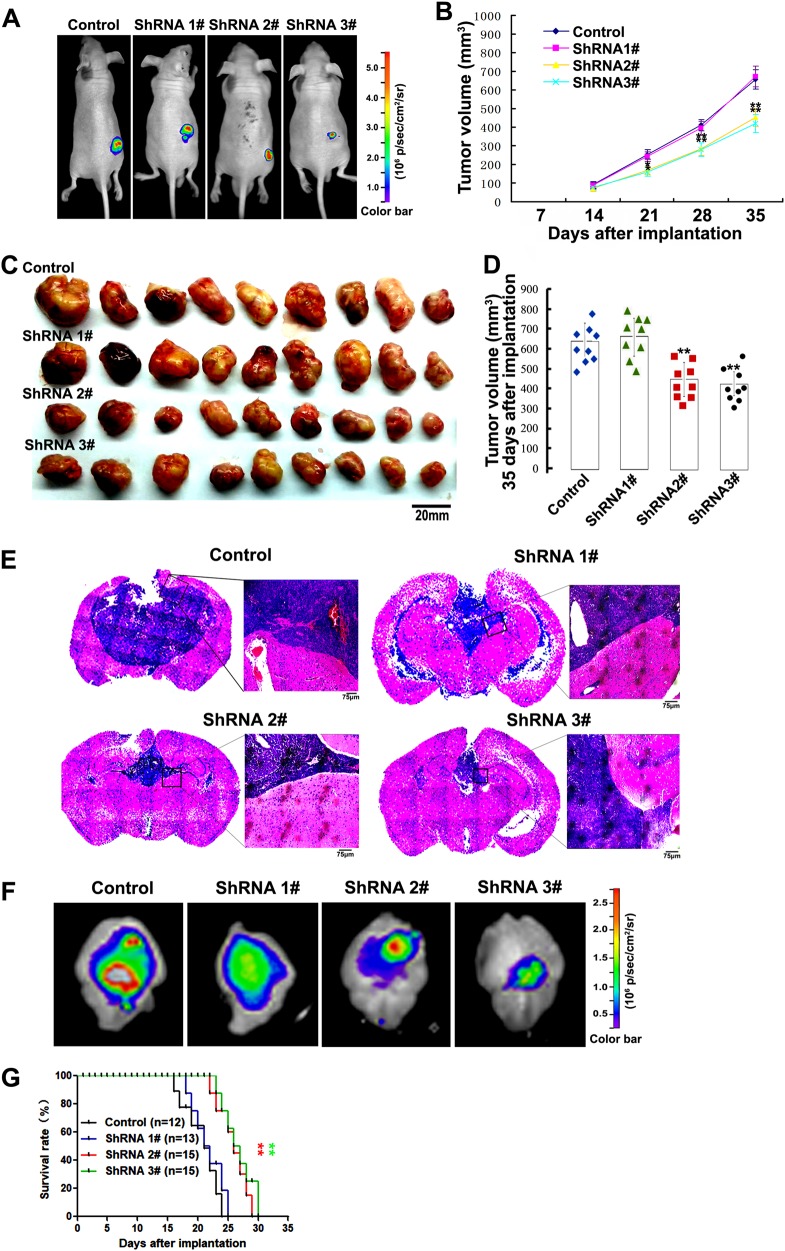
Effect of TROY knockdown on glioma development. (**a**) Subcutaneous glioma model mice induced by U87 stable transfectants (2 × 10^6^) were imaged by IVIS. The U87 cells stably expressing scramble vector were the control. GFP signal in nude mice bearing xenografted tumors were presented. (**b**) The growth curves of tumors (*n* = 9 for each group). The *x*-axis represents time after glioma cells injection. The *y*-axis represents volume of tumors (*V* = *L* × (*W*)2/2). (**c**) Subcutaneous tumors harvested from nude mice 5 weeks post xenograft (*n* = 9). (**d**) Scatter diagram of individual tumor volume 35 days after implantation. Each symbol represents a single tumor. (**e**) H&E staining of brain sections from orthotopic glioma model mice 20 days after implantation with U87 stable transfectants (5 × 10^5^). (**f**) The brains harvested from orthotopic glioma model mice 20 days after implantation were imaged by IVIS. (**g**) Overall survival were analyzed through Kaplan–Meier and a two-sided log-rank test. **P *<* 0.01*vs* control

We next used xenografted glioma models mice to examine the roles of TROY in glioma development. The tumors were detected by IVIS after subcutaneous implantation of the glioma cells stably expressing different shRNAs (Fig. 2a) and tumor volumes were evaluated every weeks (Fig. 2b). The results showed that the inhibition of TROY expression significantly slowed the development of tumor. 35 days post injection, the subcutaneous tumors were isolated from nude mice. The tumor of U87 cells stably expressing shRNA2# or shRNA 3# were observed smaller than those of the control or shRNA1#-transfected cells (Fig. 2c). Nine mice were quantified each group. The mean tumor volumes were 654.2 mm^3^ for the control cells, 671.5 mm^3^ for the shRNA1# (P > 0.05), 432.2 mm^3^ for the shRNA2# (*P* = 0.008), and 419.1 mm^3^ for the shRNA3# (*P* = 0.003), respectively (Fig. 2d).

Next, we performed the intracranial models assay as previously described [25 26]. days post implantation, H&E staining of the coronal sections of brains showed that the tumor areas from the shRNA2#- and shRNA3#-transfected cells were smaller than the control or the shRNA1#-transfected (Fig. 2e). We isolated the mice brains from the intracranial models 20 days post injection. The brains engrafted with glioma cells were detected by in vivo imaging system (Fig. 2f). In the intracranial model (Fig. 2g), mice engrafted with shRNA 2#- or 3#-transfected cells showed longer survival time compared with those from the control- or shRNA 1#-infected cells (mice number/each group were from 12 to 15). Altogether, these results suggests that knockdown of TROY suppresses human glioma development in xenografted glioma models.

### **TROY Regulates Proliferation of Glioma Cells**

To investigate the mechanism by which TROY knockdown suppresses glioma cell growth, we examined if TROY was involved in regulation of U87 apoptosis cells. Firstly, TUNEL assay was performed in the glioma cells stably expressing TROY shRNAs (Supplementary Fig. S2a, b). No significant differences were observed after knockdown of TROY, suggesting that the TROY knockdown does not affect apoptosis of the glioma cells

Next, BrdU incorporation was used to examine proliferation of the glioma cells after TROY expression knockdown. As the specific ligand of TROY receptor is unknown, we tried to detect whether FBS contained may affect the growth of glioma cells. Thus, we used the culture media without FBS as the control to detect the effect of TROY knockdown on proliferation in U87. The percentage of BrdU-positive cells was calculated to evaluate the proliferation. The significant reduction of proliferative glioma cells were observed in shRNA2# and 3#, compared with those in the control and the shRNA1# group with FBS exposure (Supplementary Fig. S2c, d). In contrast, among cells cultured in FBS-free medium, no difference in the ratio of the proliferation was observed (Supplementary Fig. S2e, f). This data suggests that the TROY knockdown inhibits the proliferation of the glioma cells after FBS exposure.

### Knockdown of TROY Induces G1-S Arrest in U87

To explore the role of TROY inhibition in glioma cell line cell cycling, the flow cytometry was used to examine the cell phases of U87 cells after TROY knockdown. The cells in the G1 phase was significantly higher in the U87 cells stably expressing TROY shRNA2# and shRNA3# than those in the controls or shRNA1# when the cells were cultured in 5% FBS medium (Supplementary Fig. S3a, b). Moreover, a significant reduction in the S phase was observed in U87 cells stably expressing TROY shRNA2# and shRNA3# compared with the controls and shRNA1# group. However, when the cells were cultured in FBS-free medium, no significant difference was found either in the G1 phase or in the S phase (Supplementary Fig. S3c, d). The data suggest knockdown of TROY expression induces G1-S arrest in the glioma cells.

### Identification of TROY Binding Proteins in Glioma Cells

Although TROY has been identified as a member of TNFR superfamily [10, 11], the downstream effects of TROY receptor binding is not well elucidated. To deepen an insight into the downstream signaling of TROY, we performed a proteomic analysis of the binding partners of TROY-ICD. The intracellular domain of TROY was used to pull down the binding candidates from rat brain lysate. The precipitations were then subjected to two-dimensional electrophoresis followed by TOF mass spectra analysis. Among the potential binding proteins, we selected three candidates (RKIP, prohibitin and RhoGDIα) to further investigate their interactions with TROY given prior reports of involvement of these proteins in glioma development [27–29].

To investigate if TROY forms a complex with these-candidates, a co-immunoprecipitation assay was performed. HA-tagged TROY was co-expressed with GFP-tagged RKIP or GFP-tagged prohibitin in HEK293T cells. Both GFP-RKIP and GFP-prohibitin were found to be immunoprecipitated with HA-TROY and vice versa (Supplementary Fig. S4a–d). The binding of the candidates with TROY were confirmed by GST pull down. As shown in Supplementary Figure S4e, f, GFP-tagged RKIP or prohibitin were precipitated by GST-TROY-ICD.

**Fig. 3:**
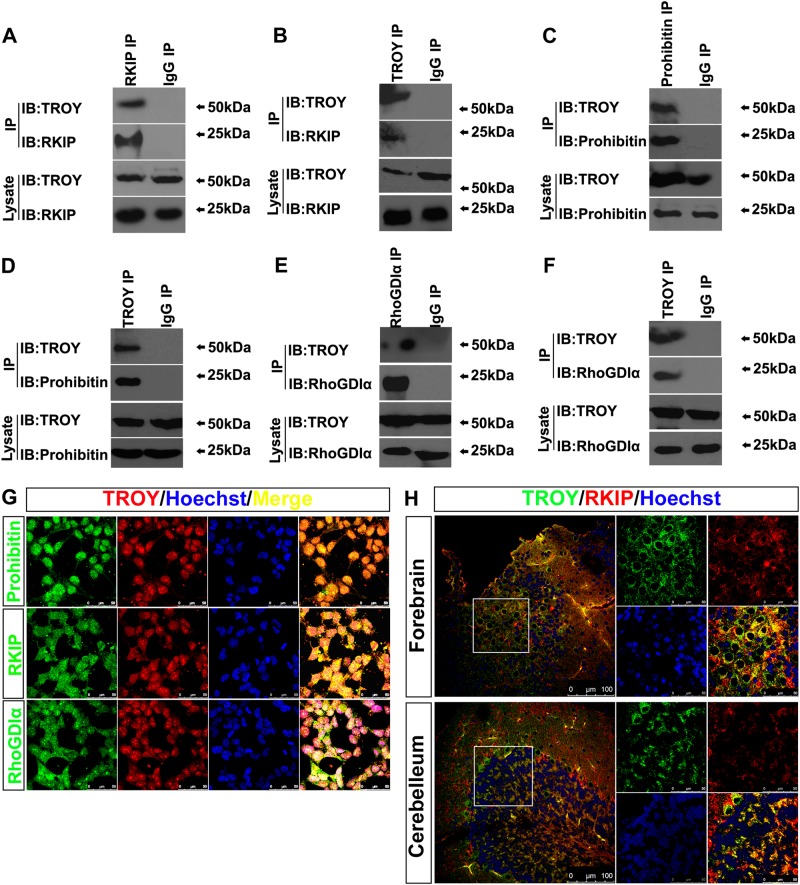
The interactions of candidates with TROY in glioma cells. (**a**-**f**) The immunoprecipitation of endogenous RKIP, Prohibitin or RhoGDIα with TROY in U87 glioma cells. The rabbit IgG was the immunoprecipitation control. (**g**) The colocalization (*yellow*) of TROY (*red*) with RKIP, Prohibitin or RhoGDIα (*green*) in U87 cell lines. (**h**) Immunohistochemistry showed the colocalization (*yellow*) between TROY (*green*) and RKIP (*red*) in the rat brain sections

Moreover, in the lysates of the cultured U87 cells, the endogenous interactions of TROY with RKIP, prohibitin, or RhoGDIα were detected. The data suggest that TROY interacts with RKIP, prohibitin and RhoGDIα in glioma cells (Fig. 3a–f). We also examined if TROY and its binding-partners were co-localized in the glioma cells. TROY and the binding partners were observed to be co-expressed in the glioma cells through immunocytochemical staining (Fig. 3g). Furthermore, immunohistochemistry showed that TROY was widely co-expressed with RKIP in rat brain (Fig. 3h).

### TROY/ RKIP Interaction is Regulated by FBS Exposure

**Fig. 4:**
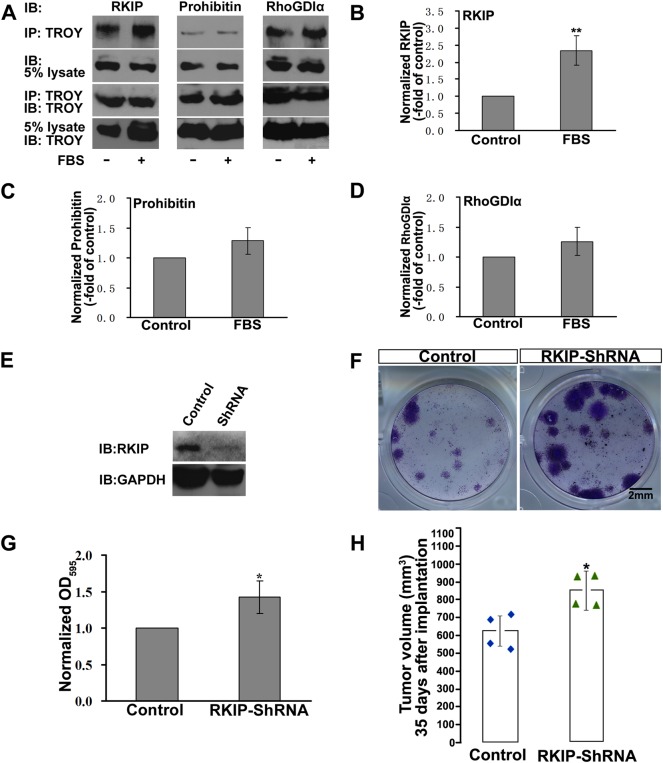
The interaction between RKIP and TROY and the effect of RKIP knockdown on glioma development. (**a**) Interaction between TROY and its binding partners in the U87 cells after FBS exposure. The cells were deprived of FBS for 6 h before exposure. The U87 cells without FBS exposure were the control. (**b-d**) The band intensities of *a* are quantified. The quantification of the precipitates are normalized to those of lysates. The treated group are normalized to the control. (**e**) RKIP protein expression in U87 stable cell lines transfected with RKIP-shRNAs or scramble vector (Control). GAPDH was an internal standard. (**f**) U87 colonies expressing RKIP-shRNAs or scramble vector (Control) were presented. (**g**) Quantification of *f*. OD595 values of RKIP-shRNAs groups are normalized to the control. *P *<* 0.05. (**h**) Scatter diagram of individual tumor volume 35 days after implantation (*n* *=* 4 for each group). Each symbol represents a single tumor. *P *<* 0.05

The previous results have showed that the TROY knockdown inhibited proliferation and induced an apparent G1/S arrest only in the presence of FBS exposure. Thus, we examined the interaction of TROY with RKIP, prohibitin, and RhoGDIα in glioma cells after FBS exposure. As shown in Fig. 4a–d, the interaction of TROY with RKIP was significantly enhanced after FBS exposure in the glioma cells. However, the FBS treatment did not affect the association of TROY with prohibitin or RhoGDIα. This suggests that the TROY/RKIP interaction is regulated by the FBS exposure.

It have been reported that RKIP play roles in several types of cancer [30–34]. Loss of RKIP accelerates cell migration and proliferation of human hepatoma cells [31]. We then evaluated whether RKIP knockdown affects the tumor volume of glioma in our in vitro and in vivo systems. We synthesized shRNAs specifically targeting human RKIP as the previous report [35]. U87 cells stably expressing RKIP shRNA was constructed. Scrambled shRNA was used as the control. The RKIP levels in U87 were significantly reduced by shRNA (Fig. 4e). In colony formation assays, transfection of RKIP shRNA markedly promoted the clonogenic proliferative ability (Fig. 4f). Quantification of the colonies show that RKIP-shRNA substantially increased the OD595 value of the colonies (P = 0.02) (Fig. 4g). We next established subcutaneously xenografted glioma models to examine the role of RKIP in glioma development. 35 days post implantation of the glioma cells stably expressing RKIP-shRNA, the subcutaneous tumors were harvested from mice. The tumor sizes formed by the U87 cells stably expressing RKIP shRNA were larger than those formed by the control cells. The mean tumor volume formed was 612.7 mm^3^ for the control cells (*n* = 4), and 851.4 mm^3^ for the RKIP-shRNA group (*n* = 4, *P* = 0.03 vs control) (Fig. 4h), respectively.

### TROY is Required for NF-κB Activation

To decipher downstream signaling mechanism of TROY, we evaluated if TROY knockdown affects activation of ERK1/2, Akt, JNK, P38. All of those have been shown to be crucial for the development of glioma [4, 9, 36]. As shown in Supplementary Figure S5a, the TROY knockdown did not affect the protein levels of activated ERK1/2, Akt, JNK or P38. As Protein kinase C can phosphorylate RKIP via regulation of the Raf/MAP kinase signaling cascade [37], we also examined whether TROY knockdown affects levels of RKIP phosphorylation. No significant alteration of phosphorylated RKIP was observed after the TROY knockdown (Supplementary Fig. S5b).

Numerous studies reported NF-κB activation is involved in oncogenesis [38, 39]. TROY overexpression induces NF-κB activation [10]. In this study, U87 cells stably expressing TROY shRNAs were transiently transfected with a NF-κB-luciferase reporter to detect the effect of TROY knockdown on NF-κB activation. As shown in Supplementary Figure S5c, the TROY knockdown significantly decreased NF-kB luciferase activity (for shRNA2#, *P* = 0.04 vs control; for shRNA3#, *P* = 0.02 vs control), compared with that for the control and shRNA1#.

### TAT-TROY (234–371 aa) Protein Disrupt the Direct Interaction between TROY and RKIP

**Fig. 5:**
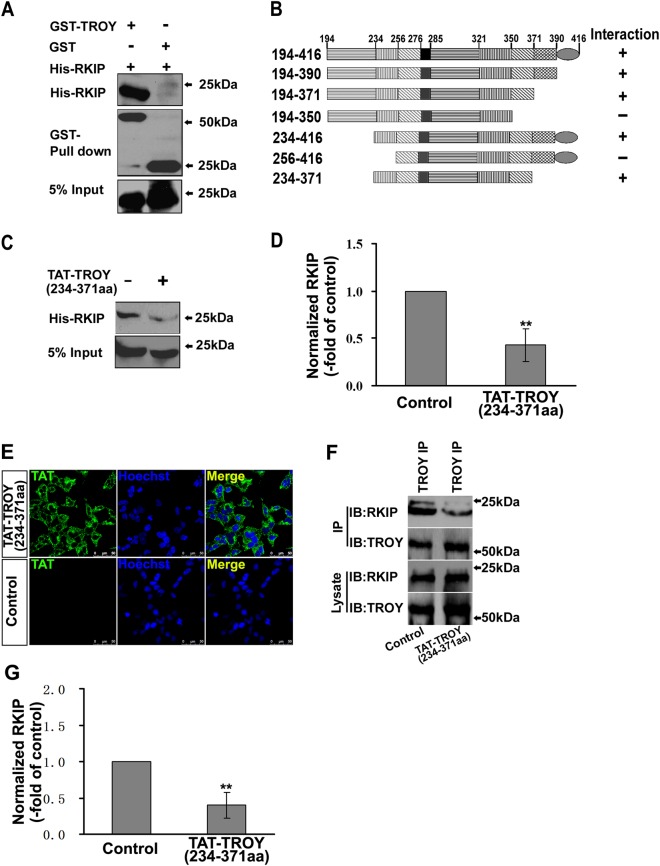
TAT-TROY (234–371 aa) protein disrupts the interaction between TROY and RKIP. (**a**) Direct interaction of RKIP with TROY by GST pull-down assay. (**b**) Schematic representation of truncation mutants of HA-TROY and their interaction with GFP-RKIP in co-transfected HEK293T cells. (**c**) The direct interaction between GST-TROY and His-RKIP were inhibited after incubation with TAT-TROY (234–371 aa). (**d**) The band intensities of *c* are quantified. The quantification of the precipitates are normalized to those of lysate. The treated group are normalized to the control. (**e**) TAT-mediated internalization. U87 cells were incubated with 0.2 μM of TAT-TROY (234–371 aa) or the PBS (control) for 20 min at 37℃. TAT fusion protein was labelled by anti-TAT antibody (*green*). (**f**) The interaction of RKIP/TROY in U87 cells was significantly inhibited after TAT-TROY (234–371 aa) protein treatment. (**g**) The band intensities of *f* are quantified. The quantification of the precipitates are normalized to those of lysates. The treated group are normalized to the control. **P *<* 0.01

We then performed a GST pull-down assay to evaluate if TROY binds RKIP directly. Consistent with the above findings, GST-TROY-ICD interacts with His-RKIP (Fig. 5a). This indicates that TROY binds RKIP directly. To map the binding region of TROY interacting with RKIP, a co-IP assay was performed with GFP-RKIP and HA-tagged deletion mutants of TROY-ICD. As shown in Fig. 5b, TROY (234–371 aa) was crucial for mediating the interaction between TROY and RKIP. We further examined whether TROY (234–371 aa) could disrupt the interaction between TROY and RKIP. We prepared TROY (234–371 aa) protein tagged with TAT peptide. The GST-TROY-ICD and His-RKIP fusion proteins were incubated with the TAT protein (10 μM). Then, the GST pull-down assay was performed. RKIP bound to TROY was remarkably decreased when the TAT protein was applied (Fig. 5c, d), suggesting that the TAT-TROY (234–371 aa) protein can disrupt the interaction of TROY/RKIP in vitro.

To confirm the effect of the TAT-TROY (234–371 aa) protein on endogeneous TROY/RKIP interaction, we tested whether the TAT protein could be transported into the glioma cells. U87 glioma cells were treated by TAT protein for 20 min as previously described [1] and subjected to immunostaining with antibodies against TAT. The TAT motif facilitated the delivery of TROY (234–371 aa) protein into the cells (Fig. 5e). Next, we tested whether the TAT-TROY (234–371 aa) protein could disrupt the TROY/RKIP interaction in the glioma cells. We incubated the TAT protein (0.2 μM) with U87 glioma cells for 20 min. Cells lysates were then subjected to immunoprecipitate with anti-TROY. The TROY/RKIP interaction in the glioma cells was significantly reduced by applying the TAT-TROY (234–371 aa) protein (Fig. 5f, g).

### Disruption of TROY/RKIP Interaction Slows the Glioma Growth

**Fig. 6:**
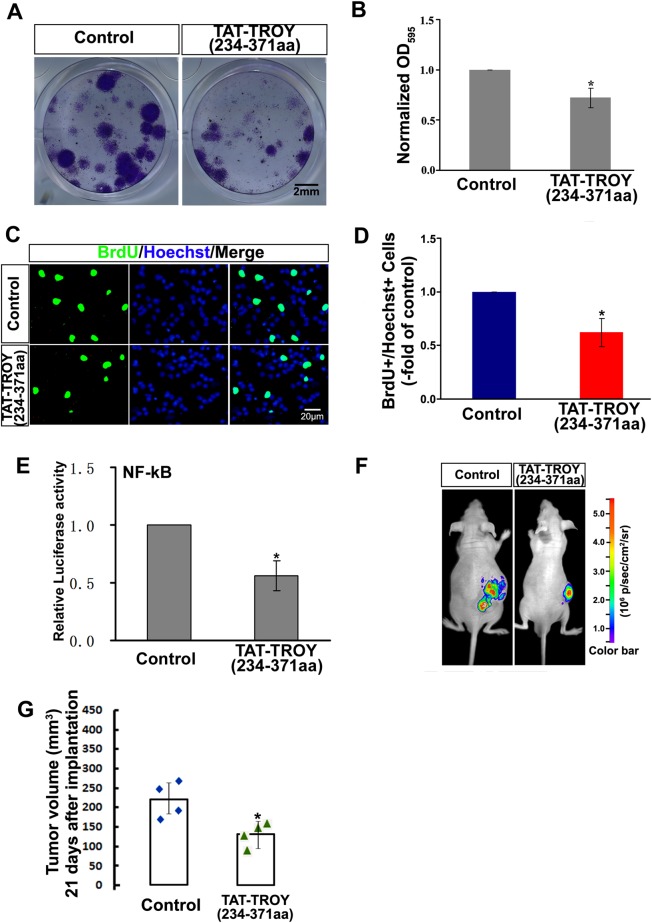
Effect of TAT-TROY (234–371 aa) on the development of glioma. (**a**) U87 colonies incubated with TAT-TROY (234–371 aa) protein, or TAT (Control) were presented. (**b**) Quantification of U87 colonies. OD595 values of the group incubated with TAT-TROY (234–371 aa) protein are normalized to that of the control group. (**c**, **d**) TAT-TROY (234–371 aa) protein slowed U87 proliferation when FBS exposure. The proliferated cells were detected by BrdU staining (*green*). (**e**) The activation of NF-kB in U87 cells were attenuated after TAT-TROY (234–371 aa) treatment. (**f**) Subcutaneous glioma model mice induced by U87 cells (2 × 10^6^) were imaged by IVIS. GFP signal in nude mice bearing xenografted tumors were presented. Mice were treated with TAT-TROY (234–371 aa) (i.p., every 2 days, 2 mg/kg/injection). The group treated by TAT was the control. (**g**) Scatter diagram of individual tumor volume 21 days after implantation (*n* = 4 for each group). Each symbol represents a singl tumor. *P *<* 0.05

To detect the effect of the TAT-TROY (234–371 aa) protein on the glioma cells, colony formation assays were performed. Incubation with the TAT-TROY (234–371 aa) protein significantly inhibited the clonogenic ability of glioma cells (Fig. 6a). Quantification of the colonies show that the TAT-TROY (234–371 aa) incubation substantially decreased OD595 value of the colonies (*P* = 0.03) (Fig. 6b).This data indicates that TAT-TROY (234–371 aa) protein appears to slow glioma cell growth.

We then examined the effect of TAT-TROY (234–371 aa) protein on the proliferation of the glioma cells. The proliferation was significantly decreased in the TAT-TROY (234–371 aa) protein-incubated U87 glioma cells, compared with that of the control incubated with PBS in 5% FBS medium (*P* = 0.006 vs control) (Fig. 6c, d). This finding suggests that the TAT-TROY (234–371 aa) protein inhibits the proliferation capacity of the glioma cells after FBS exposure in vitro.

We also examined the effect of TAT-TROY (234–371 aa) on NF-κB activation. The U87 with a NF-kB-luciferase reporter were incubated with TAT-fused protein (0.2 μM) for 20 min. The incubation with TAT-TROY (234–371 aa) protein significantly reduced the luciferase activity in the U87 cells (*P* = 0.02) (Fig. 6e).

We next examined whether a disruption of the TROY/RKIP interaction using the TAT-TROY (234–371 aa) protein treatment in the glioma cells could affect the glioma development. The mice subcutaneously injected with the U87 cells stably expressing GFP were treated with the TAT-TROY (234–371 aa) protein intraperitoneally (i.p.) as previously described [40]. We found that systemically applied TAT-TROY (234–371 aa) protein significantly reduced the glioma growth in subcutaneously xenografted glioma models mice 21 days after implantation (Fig. 6f). The tumor volume in the group treated with the TAT-TROY (234–371 aa) protein was 141.6 mm^3^ (vs 218.2 *P* = 0.04 vs control (Fig. 6g).

### Knockdown of TROY or Disruption of TROY/RKIP Interaction Alleviates the Patient-derived Xenografts Growth

We established GBM 06 as PDCs and constructed orthotropic xenograft models to validate the previous data. We constructed TROY- or RKIP-shRNA-expressing patient-derived stable clones. The stable cells were co-expressed with firefly luciferase as marker. The shRNA blank vector were used as the control. These patient-derived stable cells were seeded into culture wells to examine their colony formation in vitro. The growth of PDCs in TROY-shRNA-expressing groups were diminished significantly. Conversely, colony formation in RKIP-shRNA-expressing groups was increased compared with the control (Supplementary Fig. S6a, b). Similarly, colony formation was slower after the PDCs incubated with TAT-TROY (234–371 aa) protein (Supplementary Fig. S6c, d). These results indicate that the growths of PDCs are alleviated by TROY knockdown or disruption of TROY/RKIP interaction and increased by RKIP knockdown.

**Fig. 7:**
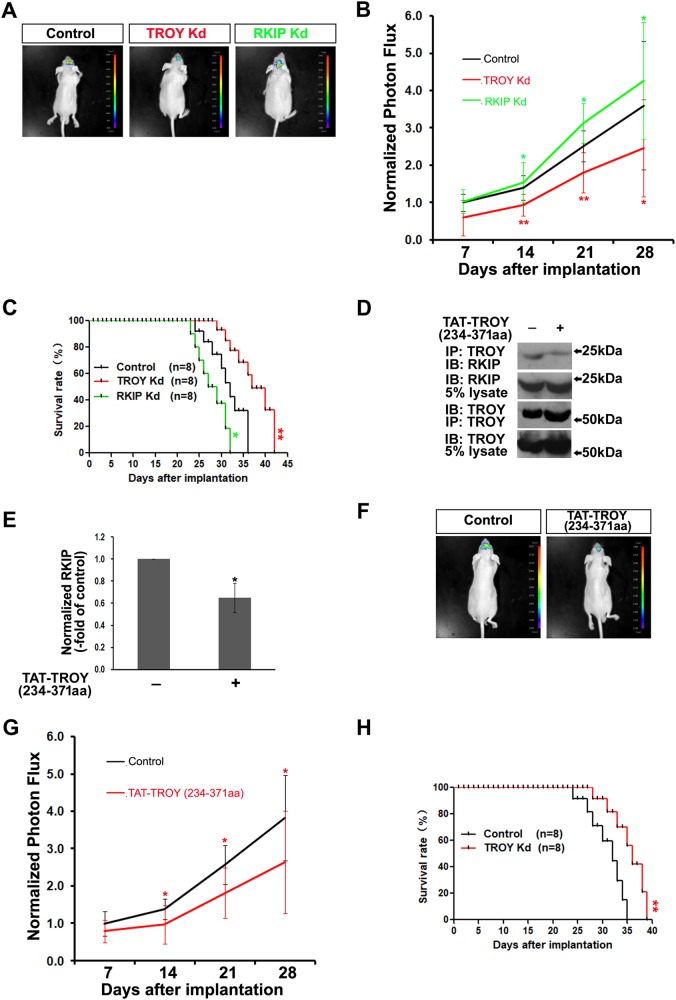
Effect of TROY-, RKIP-knockdown, and TAT-TROY (234–371 aa) protein on the development of glioma in a orthotopic model implanted by PDCs. (**a**) The nude mice intracranially implanted with PDCs stable transfectants (5 × 10^5^) were photographed by IVIS 28 days post implantation. (**b**) The relative luciferase signal captured using IVIS in each tumor at different time points were normalized to the control (Scramble vector). *n* = 8 for each group. (**c**) Survival were analyzed by Kaplan–Meier and a two-sided log-rank test. (**d**) The interaction of RKIP with TROY in PDCs was significantly inhibited after treated by TAT-TROY (234–371 aa) protein. (**e**) The band intensities of *d* are quantified. The quantification of the precipitates are normalized to those of lysates. The treated group are normalized to the control. (**f**) The nude mice intracranially implanted with PDCs (5 × 10^5^) were photographed by IVIS. Mice were *i.p*. injected with TAT-TROY (234–371 aa) protein or TAT (every 2 days, 2 mg/kg/injection), respectively. Photographs of mice 28 days post implantation were presented. (**g**) The relative luciferase signal captured using IVIS in each tumor at different time points were normalized to the control (Treated by TAT). *n* = 8 for each group. (**h**) Overall survival were analyzed through Kaplan–Meier and a two-sided log-rank test

To test the growth of orthotopic xenografts glioma models derived from PDCs in nude mice, the PDCs expressing TROY- or RKIP-shRNA were injected into the brain. The growth of implants were photographed by IVIS after the implantation every week. During the first two weeks following implantation, most of the PDCs grew slowly (Fig. 7a, b). By 14 days, those in TROY-knockdown (TROY Kd) group, but not the control, significantly alleviated the growth of implants. RKIP-knockdown (RKIP Kd) group showed the inverse results. Correspondingly, mice bearing glioma originating from TROY-Kd-infected PDCs survived longer, and the overall survival of mice xenografted with RKIP-Kd-infected PDCs were shortened significantly (Fig. 7c).

We further explored the role of disruption of TROY/RKIP interaction in PDCs-derived-models by constructing intracranial models using PDCs expressing firefly luciferase. The model mice were then *i.p*. injected with the TAT-TROY (234–371 aa) protein every 2 days. One of the PDCs-derived-glioma tissues were isolated, lysed, and then subjected to immunoprecipitation with antibodies against TROY. Both TROY and RKIP were found to be expressed in the glioma. The TROY/RKIP interaction was significantly reduced by TAT-TROY (234–371 aa) protein (Fig. 7d, e). Furthermore, the quantification of the luciferase signal using IVIS showed that TAT-TROY (234–371aa) proteins abolished the growth of PDCs-derived-orthotopic glioma (Fig. 7f, g). Meanwhile, orthotopic model mice survived longer than the control mice (*n* = 8/each group) (Fig. 7h). As such, the disruption of the TROY/RKIP interaction in the PDCs-derived-orthotopic glioma with the TAT-TROY (234–371 aa) protein appeared to inhibit the in vivo development of the xenografted gliomas.

## Discussion

Gliomas, particularly the high grade variety, are characterized by high recurrence rate, rapid growth, relative resistance to medical therapy, and a high degree of invasiveness [41]. In the present study, there was overexpression of TROY protein levels in glioma cell lines and patient-derived tumor samples. Trans et al. has previoulsy reported TROY to be upregulated in human glioma tissues and cells [22, 23]. These results taken together indicate that TROY may be a putative biomarker for human glioma.

TROY is thought to functionally connect with the apoptosis and classified as a member of TNFR family [11]. However, TROY can not bind to any of the TNF ligand family members [2] and also has a unique cytoplasmic domain, suggesting that the function of TROY may be distinct from the other TNFR members. In fact, TROY possesses various functions distinct from apoptosis. For instance, TROY mediates neurite outgrowth inhibition in neurons via activating RhoA signaling [12, 13]. In certain proliferating tissues such as rostral migratory, subventricular precursor cells and astrocytes, TROY expression is enriched, leading to the possibility that it may regulate cell proliferation or play roles in gliogenesis in the adult CNS [4, 14, 42–44]. In our study, overexpression of TROY in glioma tissues was identified, implying potential roles of TROY in tumorigenesis. Moreover, we found the inhibition of TROY decreased the proliferation of glioma cells and induced G1/S arrest of the glioma cells. TROY inhibition suppressed both the clonogenic formation ability of the glioma cells and the glioma tumor volume growth in xenografted models. Of note, Tran et al. found that overexpression of TROY had not influence in the proliferation of glioma cells [23]. In their system, TROY was expressed in T98G cells and the proliferation was examined by Alamar Blue assay. In our system, we knocked down TROY expression in U87 cells and detected the effect of endogenous knockdown of TROY on the proliferation via BrdU incorporation assay. Thus, we hypothesize that the apparent discrepancy may be that the high endogenous TROY protein levels in glioma cells may cause functional saturation.

As an orphan receptor, the specific ligand of TROY has not been identified yet. It has been reported that TROY can bind to lymphotoxin-alpha and activate NF-κB-mediated transcription [45]. However, lymphotoxin-alpha is primarily secreted by lymphocytes and TROY is not expressed in lymphoid tissues, suggesting that the physiological ligand of TROY needs to be explored further. Interestingly, we found that the effect of TROY knockdown on cell cycles and proliferation was dependent on FBS exposure. Cancer cells can sustain proliferative signaling through several ways. They can stimulate the proliferation of themselves via autocrine [46] or receptor signaling be regulated by up-regulating levels of receptor proteins, rendering such cells hyper-responsive to growth factor ligands [9, 46]. Alternatively, sustaining proliferative signaling may derive from the constitutive activation of the downstream molecules. Given TROY expression was up-regulated in various types of human gliomas, we speculate high expression of TROY may render glioma cells hyper-responsive to some factors in FBS. The identity of the TROY ligand has not been elucidated and remains a key point of future investigation.

We identified that RKIP interacts with the intracellular domain of TROY. Moreover, the physical interaction of TROY/RKIP was confirmed by immunoprecipitation in both ectopic expression systems and glioma cells. RKIP has been shown to be a multifunctional protein that controls cellular growth, motility, differentiation, and tumor metastization [26–29, 45]. The loss of RKIP expression is linked to worse clinical outcomes and advanced tumor stages [34]. Interestingly, the TROY/RKIP interaction in glioma cells was enhanced by FBS exposure, while both TROY/RhoGDIα and TROY/prohibitin associations were not significantly influenced, indicating that the TROY/RKIP interaction was specific and ligand-dependent in the glioma cells. Furthermore, the disruption of the TROY/RKIP interaction in the glioma cells with the TAT-TROY (234–371 aa) protein decreased both the clonogenic proliferative ability of the glioma cells and the glioma growth in xenografted glioma models.

RKIP is reported to negatively regulate several important signaling pathways, including the NF-κB and RAF/MEK/ERK transduction [47–50]. The roles of NF-κB in oncogenesis include induction of proliferation, suppression of apoptosis, invasion and angiogenesis [38, 39, 51, 52]. NF-κB can promote cell growth [38, 53]. It had been reported that the overexpression of TROY greatly upregulated NF-κB activity [10, 45]. Here, we observed that TROY knockdown decreases NF-κB activity in glioma cells. Importantly, we saw that the disruption of TROY/RKIP interaction by TAT-TROY (234–371 aa) protein treatment could decrease the NF-κBactivity in glioma cells. These findings support a putative model whereby TROY functions as growth-promoting signaling molecule that also regulates the NF-κB pathway via interacting with RKIP.

It is known the activation of Raf/MEK/ERK pathway can inhibit NF-κB [3]. RKIP is widely reported to inhibit the Raf pathway as well [29, 45, 47, 48]. However, we did not observe significant alteration of phosphorylation in either Erk1or 2, which are the key downstream effectors of the Raf/MEK/ERK pathway, after TROY knockdown. Additionally, overexpression of TROY has been reported to increase survival through activation of Akt and NF-kB in T98G cells after serum-starved [22]. We did not find downstream activation of Akt in glioma cells after knockdown of TROY. On the basis of these data, it seemed that the TROY-mediated NF-κB activation through RKIP was independent of both Raf/MEK/ERK and Akt pathways.

**Fig. 8:**
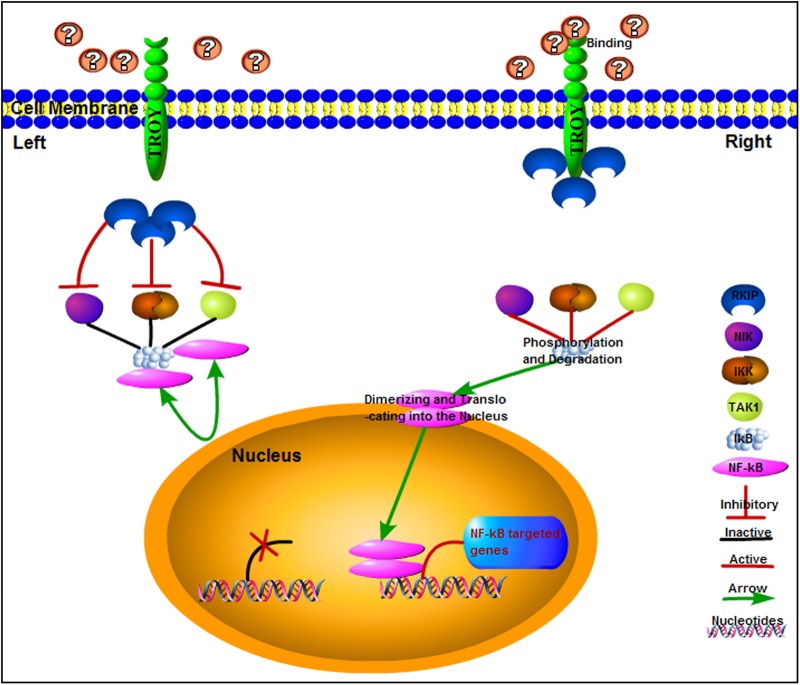
A schematic model for downstream signaling pathway of TROY/RKIP interaction in glioma RKIP interacts with upstream regulatory molecules of NF-κB (IKKα, IKKβ, NIK and TAK1) and negatively modulate activation of NF-κB (left). After FBS exposure, the RKIP are retained by TROY receptor through interaction. These retention subsequently attenuate the inhibitory effects of RKIP on the upstream active regulators of NF-κB and result in the liberation from the upstream inactive complex, IκB, which resulting in NF-κB activation. This activation can in turn lead to the downstream transcriptional activation of cell-cycle genes, promoting proliferation and cell cycles following extracellular stimulation (right)

Since TAT-TROY (234–371aa) prevents association of TROY/RKIP, and inhibits glioma growth, it is possible that RKIP was disassociated from TROY to exert the regulatory effect on downstream pathway. RKIP has been reported to interact with upstream regulatory molecules of NF-κB, including IKKα, IKKβ, NIK and TAK1, to inhibit NF-κB activation [48, 54]. Our observations that TROY/RKIP interaction was regulated by FBS stimulation naturally leads to speculation that NF-κB activation by TROY expression may be attributable, at least partly, to RKIP retention by TROY. The retention of RKIP by TROY receptor may attenuate the inhibitory effects of RKIP on the upstream active regulators of NF-κB and result in the liberation of it from the upstream inactive complex, IκB, leading to NF-κB activation. The activation in turn may lead to the downstream transcriptional activation of cell-cycle genes, promoting proliferation and cell cycles, following extracellular stimulation (as shown in Fig. 8). This retention of RKIP by TROY upon extracellular FBS exposure may result in some alterations of downstream signaling involved by RKIP. The evidence showing that the disruption of the TROY/RKIP interaction in glioma cells decreased NF-κB activity supports this speculation. However, the detail underlying mechanisms need to be further detected in the future.

## Materials and Methods

### Animal Studies

The animal experiments were approved by Committee on Animal Care. The BALB/c nude mice at 3–4 weeks of age were used in xenograft tumor model. The ectopic and orthotopic tumor growth assays were performed as previous reports [25, 26]. The details were described in the Supplemental Information.

### Pathological Tissues

We obtained human glioma tissue samples from RenJi Hospital. Samples were reviewed by the pathologists at the Hospital. The normal tissues were isolated from patients with cerebral hernia or cerebral hemorrhage who underwent decompressive evacuation of normal brain parenchyma. Human samples were used after approved by the ethics committees of RenJi hospital.

### Statistical Evaluation

Data from two groups were compared by unpaired two-tailed t tests and those from three groups were analyzed by ANOVA. Data are shown as mean ± SD. Overall survival were analyzed by Kaplan–Meier followed by a two-sided log-rank test. For comparisions, *P < 0.05, **P < 0.01 were considered significant from three independent experiments.

## Electronic supplementary material

Supplementary Data

Supplementary Figure S1

Supplementary Figure S2

Supplementary Figure S3

Supplementary Figure S4

Supplementary Figure S5

Supplementary Figure S6
