# Supplementary material for: TROY interacts with RKIP to promote glioma development
Source: Oncogene. 2018 Oct 18;38(9):1544–59. doi: 10.1038/s41388-018-0503-x (PMC6372479; doi:10.1038/s41388-018-0503-x)
Supplement: Supplementary file 1 — Supplementary Data [file 41388_2018_503_MOESM1_ESM.docx]

**Supplementary Information**

**TROY interacts with RKIP to promote glioma development**

Xiujie Liu, Yinghui Bao, Wei Meng, Ping Yang, Yi An, Jie Ma, Yujie Tang, Zhigang Liu, Yan Lu, Jianfeng Zhou, Yong Zhang, Jifeng Feng, Xiaofei Gao, Zhida Su, Yingyan Pu and Cheng He

**Supplemetary Materials and Methods**

**Materials and Reagents**

The antibodies for IB4, NG2, GFAP, and glutathione S-transferase (GST) were from Millipore (Billerica, MA). The mouse antibodies against TROY, TAT, RKIP and pRKIP were from Santa Cruz Biotechnology. The antibodies against RhoGDIα, ERK1/2, pERK1/2, Akt, pAkt, P38, pP38, JNK and pJNK were from Cell Signaling. anti-HA was from Roche, Abmart or Sigma, respectively. The other antibodies were as follows: anti-GFP, from Roche; anti-prohibitin, from Neomarkers; anti-GAPDH, from Kangcheng; anti-BrdU, from Thermo. The TUNNEL kit was purchased from Roche.

***Plasmids construction***

The cDNAs for the intracellular domain of TROY (TROY-ICD) (NM_001164155; nucleotides 742-1413; amino acids 194–416) and mutants were subcloned into pKH3. cDNA of RKIP（NM_002567）, Prohibitin (NM_001281496), and RhoGDIα (NM_001185077) were subcloned into the peGFP-N2 vector, respectively. pGEX-4T_1_-TROY-ICD was subcloned to generate GST-TROY-ICD fusion proteins. cDNAs for TROY-ICD (234-371 aa) and RKIP were subcloned into the pTAT-HA (provided by Dr. Steven F Dowdy) to generate HA-TAT-TROY(234-371 aa) and RKIP proteins. TROY (234-371 aa) protein tagged with TAT peptide (YGRKKRRQRRR) would achieve intracellular delivery.^1^

***Cell Culture***

The glioma cells (U-87, A172, U-251, and T98G), including the HEK293T, were from American Type Culture Collection (ATCC, Manassas, VA). The cells were cultured in Dulbecco’s modified Eagle medium (DMEM) (Cat. No. 11995-065, Gibco), and 5% fetal bovine serum (Cat. No. 26140-079, Gibco) under 5% CO2 at 37°C. In assays of proliferation, cell cycle, and endogenous interactions after FBS exposure, the cells were deprived of FBS for 6 hours to diminish the background before stimulation. To construct U87 cells stably expressing shRNAs, shRNA vectors expressing double-stranded oligonucleotides targeting human TROY (NM_018647) were synthesized by GenePharma or GeneChem. The shRNA vectors also express EGFP or firefly luciferase. The siRNA sequences are: 1#, 5-ATCAACTCAGGATGCACTA-3, 2#, 5-TCAACGTCTTTGGATTCAA-3, 3# 5-AGGCTATTTGTCATGTAAA-3. The sequence of scramble control is: 5-TTCTCCGAACGTGTCACGT-3. RKIP shRNA sequence was designed according to previous studies,^2^ and as follows: RKIP siRNA sequences, 5’-GGTGGCGTCCTTCCGTAAA-3’. The control shRNA vector (nonsilencing) was purchased from GeneChem or GenePharma (Shanghai, China).

Patient-derived cultures (PDCs) were established from tumor tissues of patients as described previously.^3^ Briefly, tumors were dissociated into single cells by incubated in TrypLE™ Express Enzyme (Life technologies, 12604-021) for 15min. Dissociated cells were transferred to a flask coated with laminin (Sigma, L2020) after forming spheres/aggregates in culture. Cells were then maintained using NeuroCult NS-A Kit (Stem Cell Technology, 05751) supplemented with human EGF-basic (20 ng/ml) (PeproTech, AF-100-15-100), human FGF-basic (20 ng/ml) (PeproTech, 100-18B-100) and 0.2% Heparin Solution (10 ng/ml) (Stem Cell Technology, 07980). This PDCs glioblastoma multiforme 06 (GBM 06) was identified with short tandem repeat profiling and tested for mycoplasma contamination before the use. Human tissues used were approved by patients and ethnics committee of RenJi Hospital.

***Pathological Tissues***

We obtained human glioma tissue samples from RenJi Hospital. Samples were reviewed by the pathologists at the Hospital. The normal tissues were isolated from patients with cerebral hernia or cerebral hemorrhage who underwent decompressive evacuation of normal brain parenchyma. Human samples were used after approved by the ethics committees of RenJi hospital.

**Immunohistochemistry and Immunocytochemistry**

Immunohistochemistry (IHC) and Immunocytochemistry (ICC) were performed as the previous report.^4^ The animals were intracardially injected with 4% PFA under deep anaesthesia. The tissues were cut into frozen sections after fixation and dehydration. The antigens in the specimens were recognized by the corresponding primary antibodies and labelled with secondary antibodies (Jackson ImmunoResearch and Vector Laboratories，USA). The BrdU proliferation assay was performed according to the previous report.^4^ The specimens were pre-treated with BrdU (10 μM, Sigma) before harvested. The TUNEL assays (Roche) were performed as previously described.^5^ At least 500 cells were examined in these assays.

Cell growth assay was performed as previous report.^6^ For colony formation assays, 2×10^3^/well cells were seeded in the six-well plate. Two weeks later, the colonies were stained by crystal violet (0.5%). After digested with 10% acetic acid, the op­tical density of the colonies at 595 nm (OD595) were quantified.

***Glioma Growth In Vivo Assays***

Glioma growth assay in vivo was performed as previous reports.^6,7^ In the subcutaneous glioma mouse model, 2×10^6^ glioma cells suspended in DMEM (without antibiotics) were subcutaneously injected into the nude mice at 3–4 weeks of age. The length (*L*) and width (*W*) were measured. Tumor volume (*V*) was calcu­lated as follows: *L* × (*W*)2/2.

In vivo imaging was carried out according to the previous report.^7^ The mice were anesthetized followed by imaged with In Vivo Imaging System (IVIS, Xenogen，USA) . To examine luciferase signal in tumors, the mice were imaged using IVIS 10 minutes after intraperitoneal injection of 3mg of luciferin (Gold Biotech, St Louis,MO). The mice without IVIS signals 7 days post implantation were excluded from the subsequent experiments. Animal study was used a double-blind, randomized approach.

As for the intracranial glioma model, a 0.5mm hole was made 2 mm right to the midline and 1 mm anterior to the bregma of nude mice (3-4 weeks of age) brain before implantation. 5×10^5^ glioma cells were stereotactically injected into the above hole to a depth of 3-5 mm.

***GST pull-down assay***

Pull-down was performed as our previous report.^8^ GST-fused proteins were expressed and purified with Sepharose 4B beads (Amersham Biosciences). TAT-fused proteins were purified with Ni-Sepharose beads (Qiagen, Chatsworth, CA). GST proteins were incubated with lysates from cells, tissues, or purified His-RKIP proteins at 4℃. GST proteins were used as the control. Both the precipitated beads and the supernatants were prepared as the samples for the next immunoblotting analyses.

**Co-immunoprecipitation and Western Blot**

Immunoprecipitation were performed according to published report. ^4^ The lysates homogenated from tissues or cells were incubated with both IP-antibodies and Protein G-agarose beads (Roche) overnight at 4℃. Both the precipitations and the supernatants were boiled with 1×loading beffer after washing three times for the next western blot analyses. Image-Pro Plus 5.1 (IPP5.1) software was used to quantify the density of the bands.

***NF-κB-dependent Reporter Assays***

The 10^6^ cells/well were seeded, and then co-transfected with various plasmids, including a NF-κB-driven luciferase reporter or control plasmid encoding luciferase (Promega) on the next day. The activity of luciferase was scored by a luciferase reporter 36 h later (Promega, USA).

**Supplementary Figure Legends**

**Figure S1.** Effect of TROY knockdown on glioma cell growth. **(a)** Western blot of TROY levels from U87 stable cells transfected with TROY shRNAs (1#, 2#, 3#). Blank vector was an internal control. **(b)** The band intensities of a are quantified and normalized to the control. **(c)** Representative images of colonies expressing TROY shRNAs (1#, 2#, 3#), or blank vector (Control). **(d)** Quantification of colonies in *c*. OD595 values of TROY shRNAs (1#, 2#, 3#) groups are normalized to that of the control.

**Figure S2.** Effect of inhibition of TROY on the survival and proliferation. **(a)** TROY shRNA had no effect on apoptosis of U87 cells examined by TUNEL (*red*). **(b)** The quantification of ***a*** is normalized to the control. **(c, d)** TROY knockdown inhibited U87 proliferation after FBS exposure. The proliferated cells were labelled by BrdU staining (*red*). **(e, f)** The effect of TROY knockdown on the proliferation of U87 without FBS exposure. The proliferated cells were detected by BrdU staining (*red*).

**Figure S3.** Effect of TROY knockdown on the cell-cycle. **(a, b)** The effect of TROY knockdown on the cell-cycle of the glioma cells were analyzed by flow cytometry after stained by PI. Knockdown of TROY expression induced G1/S arrest in glioma cells. The distributions of cells in G1, S and G2 phases are shown. ***P* <0.01 **(c, d)** The effect of TROY knockdown on the cell-cycle of glioma cells cultured in FBS-free medium. At least 2×10^4^ cells were examined in cytometry assay.

**Figure S4.** The identification of RKIP or Prohibitin as the TROY-interacting molecules**. (a,b)** The interaction of GFP-RKIP with HA-TROY in vitro. GFP-RKIP and/or HA-TROY-ICD were expressed in HEK293T. The lysate were immunoprecipitated by corresponding antibodies as indicated. IP, immunoprecipitation; IB, immunoblot. **(c, d)** The interaction of GFP-Prohibitin with HA-TROY in vitro. **(e, f)** GST pull-down assay of GST-TROY-ICD protein with GFP-RKIP or GFP-Prohibitin in transfected HEK293T cells. GST protein was the control.

**Figure S5.** The effect of TROY knockdown on downstream signaling in U87 cells**. (a)** The phosphorylation of P38, Erk1/2, Akt, and JNK. Relative protein phosphorylation of P38, Erk, Akt, and JNK were calculated by normalization to total protein. **(b)** The phosphorylation of RKIP. Relative phosphorylation of RKIP was normalized to total protein levels. **(c)** Reporter assay analysis of NF-kB activation in U87 cells expressing TROY shRNAs (1#, 2#, 3#), or blank vector (*Control*). **P*<0.05 *vs* control.

**Figure S6.** Effect of TROY-, RKIP-knockdown and TAT-TROY (234-371aa) protein on PDCs growth in vitro. **(a)** Representative images of PDCs colonies expressing TROY shRNAs, RKIP shRNAs, or blank vector (Control). **(b)** Quantification of PDCs colonies in *a*. OD595 values of TROY or RKIP shRNAs group are normalized to that of the control group. **(c)** Representative images of PDCs colonies treated by TAT-TROY (234-371aa) protein, or TAT (Control). **(d)** Quantification of PDCs colonies. OD595 values of the group incubated with TAT-TROY (234-371 aa) protein are normalized to that of the control group. **P* < 0.05.

**Supplemental References**

1. Schwarze SR, Ho A, Vocero-Akbani A, Dowdy SF. In vivo protein transduction: delivery of a biologically active protein into the mouse. *Science* 1999; **285**: 1569-1572.
2. Trakul N, Menard RE, Schade GR, Qian Z, Rosner MR. Raf kinase inhibitory protein regulates Raf-1 but not B-Raf kinase activation.*J Biol Chem*. 2005; **280**:24931-24940.
3. Pollard SM, Yoshikawa K, Clarke ID, Danovi D, Stricker S, Russell R, et al. Glioma stem cell lines expanded in adherent culture have tumor-specific phenotypes and are suitable for chemical and genetic screens. *Cell Stem Cell* 2009; **4**; 568-580.
4. Liu X, Lu Y, Zhang Y, Li Y, Zhou J, Yuan Y,et al. Slit2 regulates the dispersal of oligodendrocyte precursor cells via Fyn/RhoA signaling. *J Biol Chem*2012; **287**: 17503-17516.
5. Yuan Y, Su Z, Pu Y, Liu X, Chen J, Zhu F,et al. Ethyl pyruvate promotes spinal cord repair by ameliorating the glial microenvironment. *Br J Pharmacol*2012; **166**: 749-763.
6. Ding X, He Z, Zhou K, Cheng J, Yao H, Lu D, et al.Essential role of TRPC6 channels in G2/M phase transition and development of human glioma. *J Natl Cancer Inst*2011; **102**: 1052-1068.
7. Ziegler DS, Wright RD, Kesari S, Lemieux ME, Tran MA, Jain M,et al. Resistance of human glioblastoma multiforme cells to growth factor inhibitors is overcome by blockade of inhibitor of apoptosis proteins. *J Clin Invest* 2008; **118**: 3109-3122.
8. Lu Y, Liu X, Zhou J, Huang A, Zhou J, and He C. TROY interacts with Rho guanine nucleotide dissociation inhibitor alpha (RhoGDIalpha) to mediate Nogo-induced inhibition of neurite outgrowth. *J Biol Chem*2013; **288**: 34276-34286.
