# Supplementary figures and images for: TROY interacts with RKIP to promote glioma development
Source: Oncogene. 2018 Oct 18;38(9):1544–59. doi: 10.1038/s41388-018-0503-x (PMC6372479; doi:10.1038/s41388-018-0503-x)

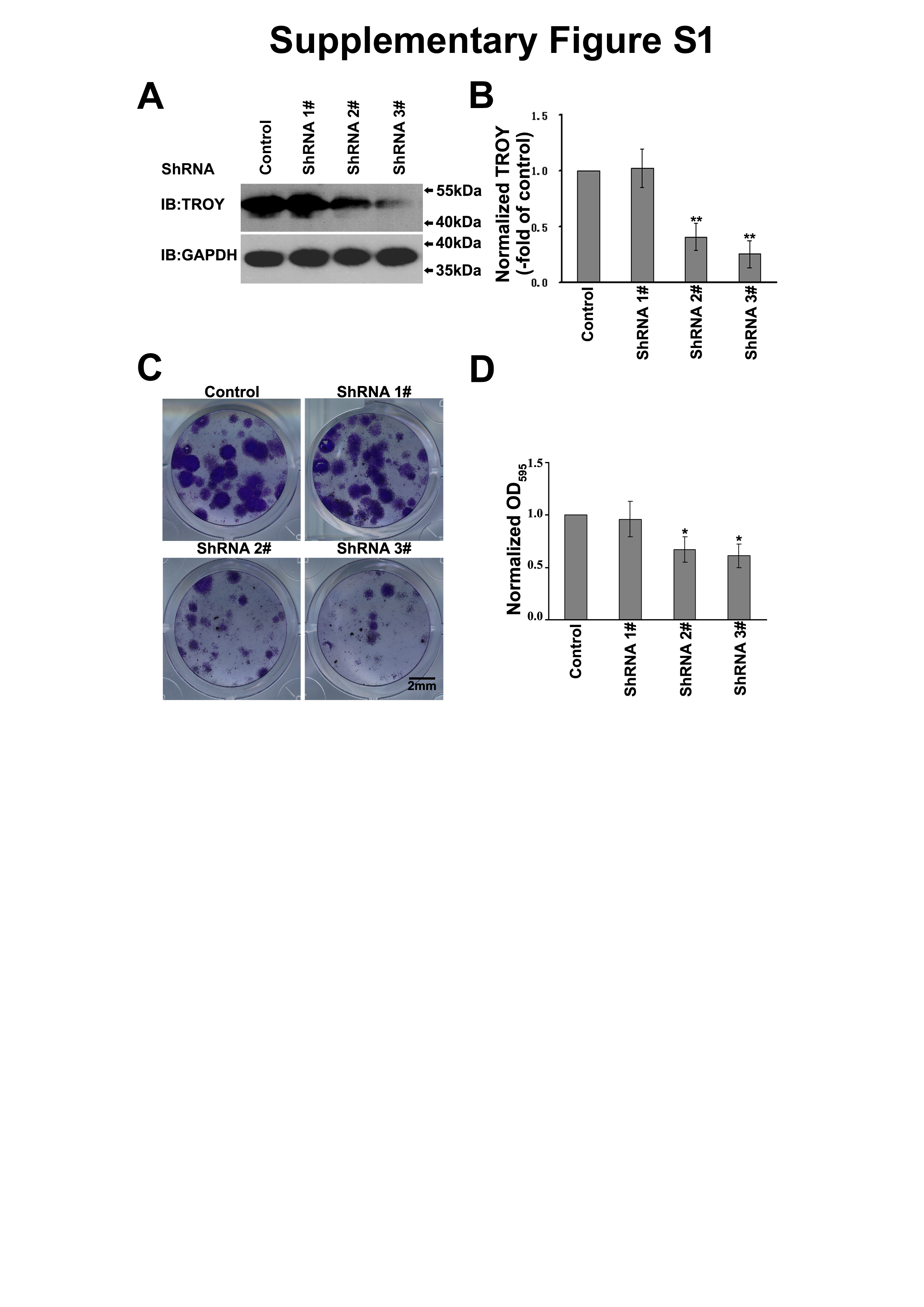

Supplement: Supplementary file 2 — Supplementary Figure S1 [file 41388_2018_503_MOESM2_ESM.tif]

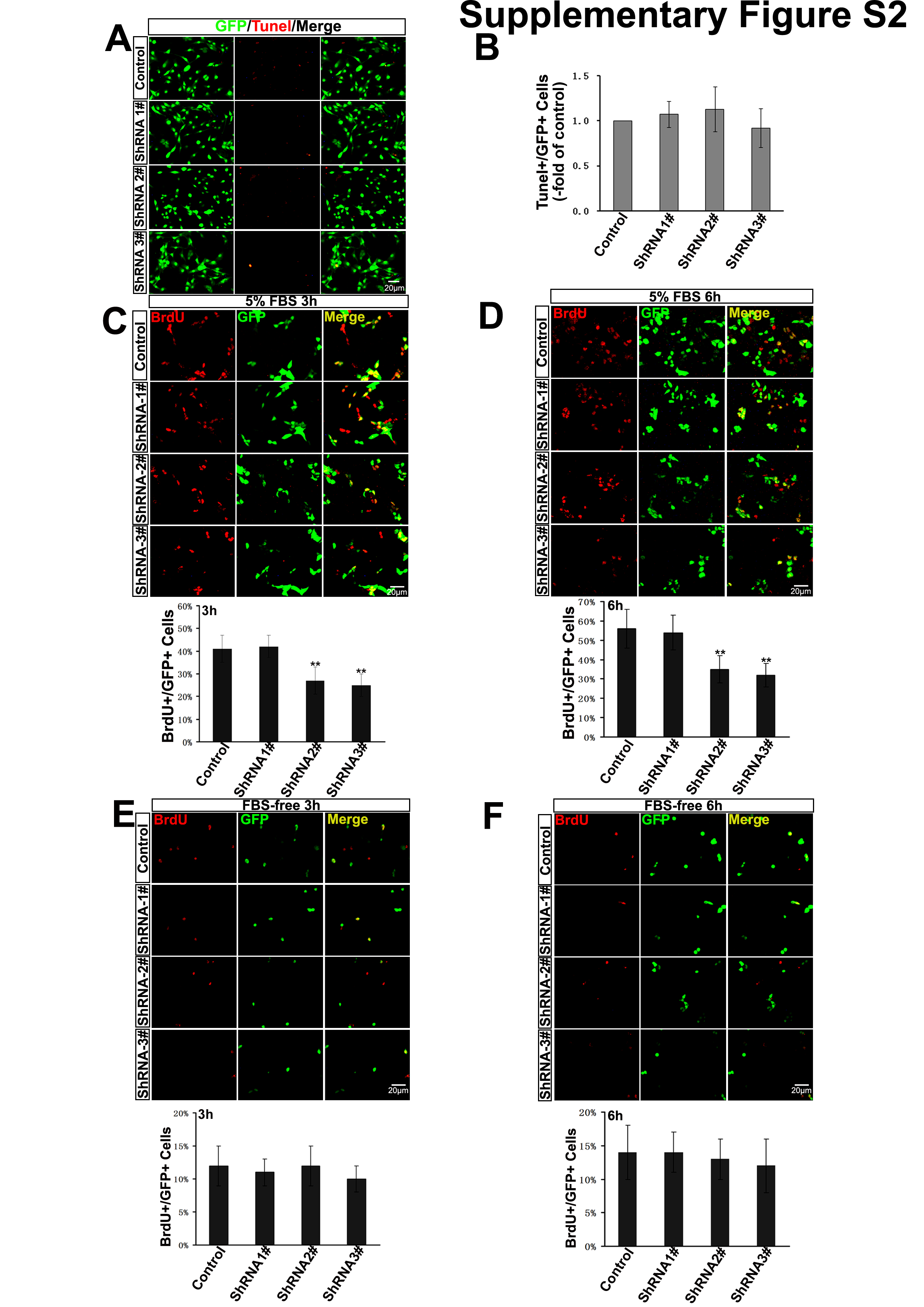

Supplement: Supplementary file 3 — Supplementary Figure S2 [file 41388_2018_503_MOESM3_ESM.tif]

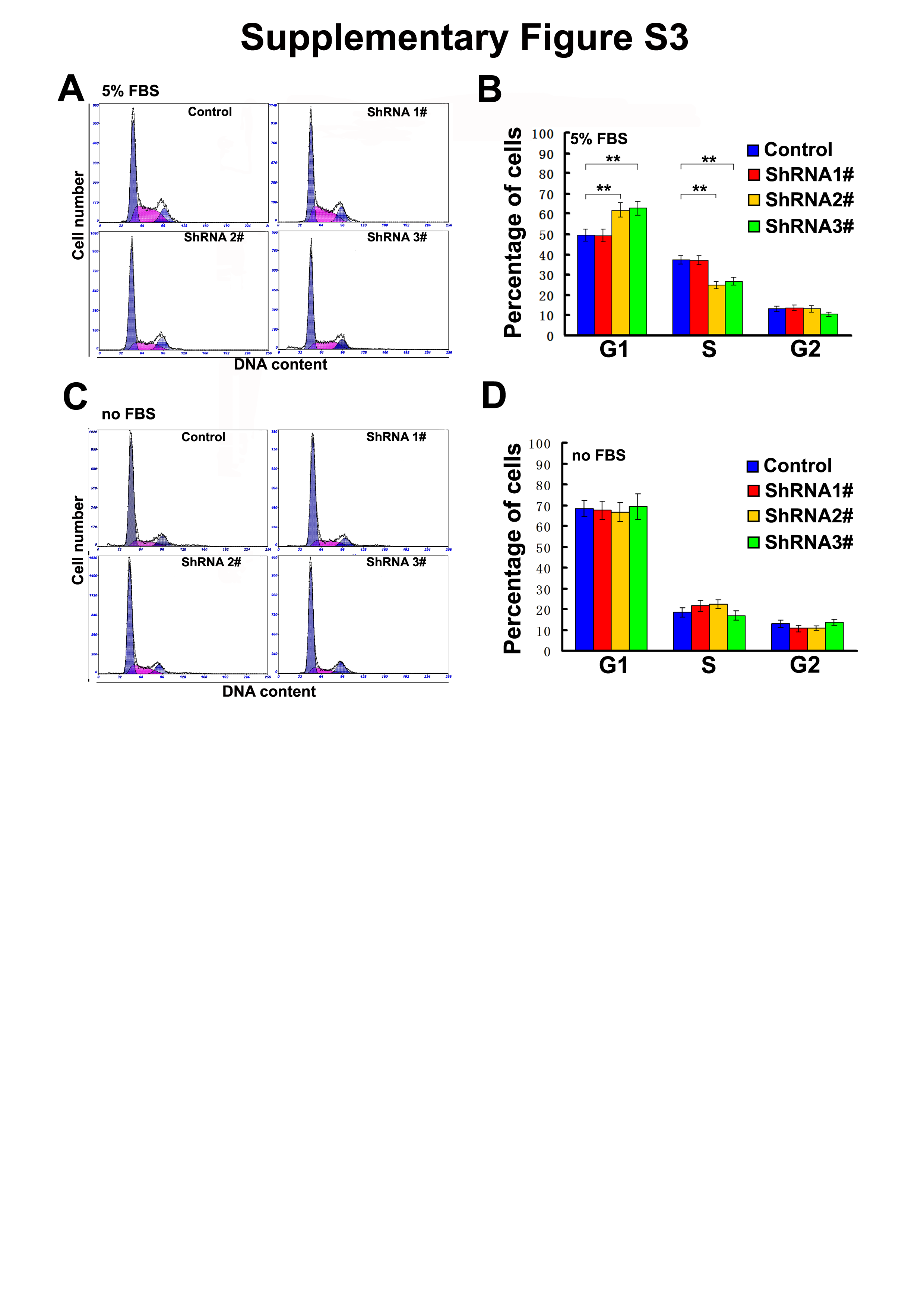

Supplement: Supplementary file 4 — Supplementary Figure S3 [file 41388_2018_503_MOESM4_ESM.tif]

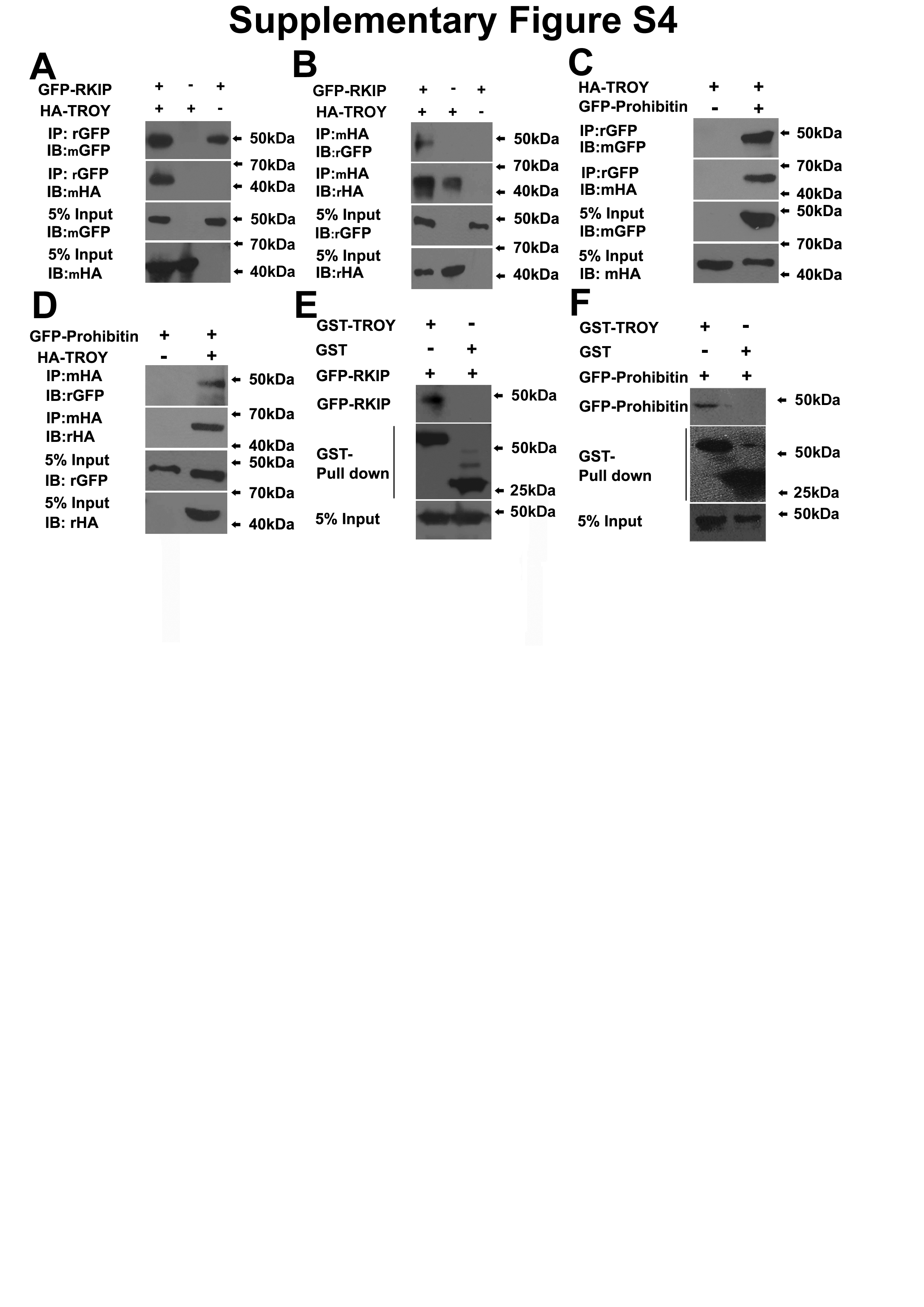

Supplement: Supplementary file 5 — Supplementary Figure S4 [file 41388_2018_503_MOESM5_ESM.tif]

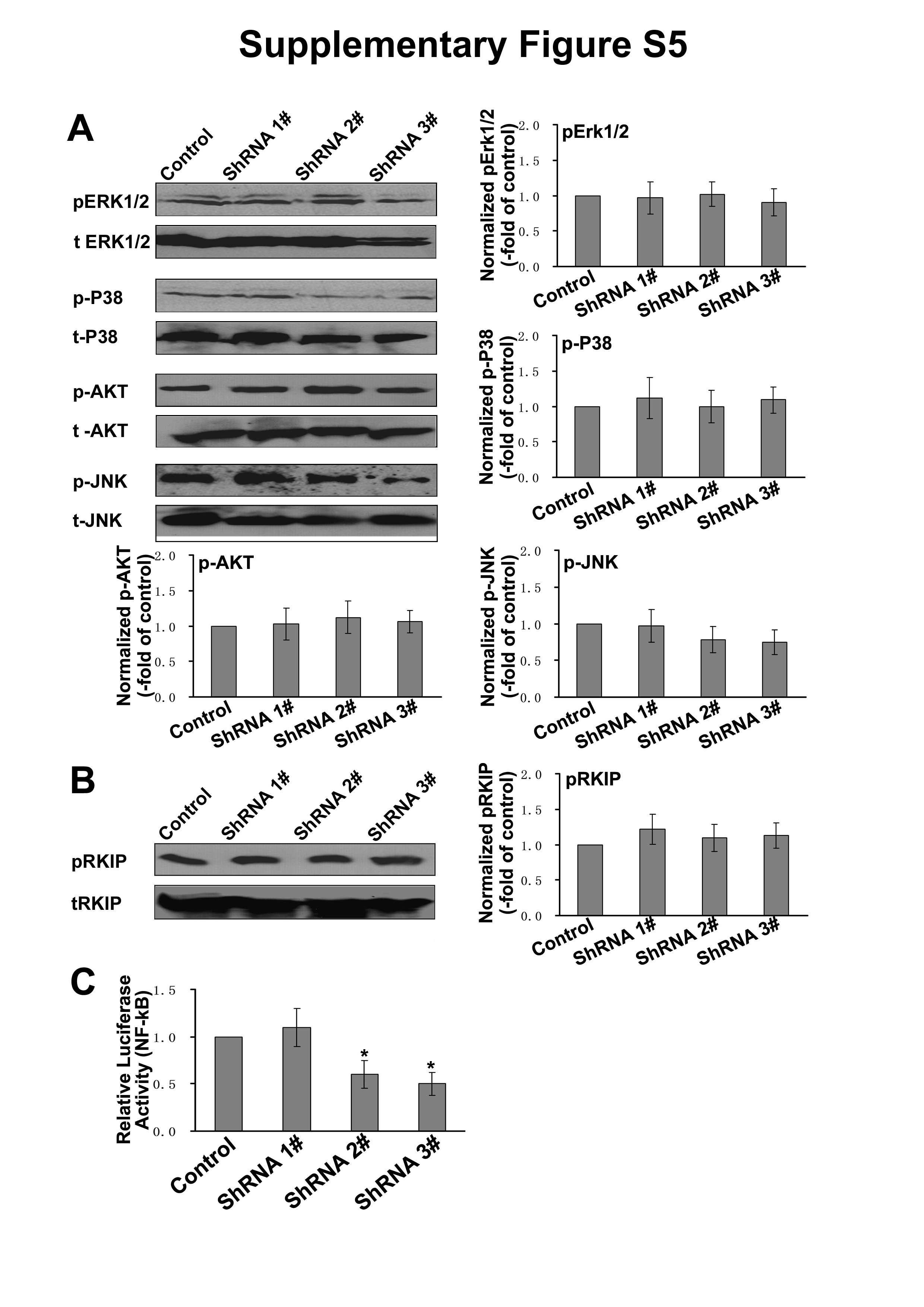

Supplement: Supplementary file 6 — Supplementary Figure S5 [file 41388_2018_503_MOESM6_ESM.tif]

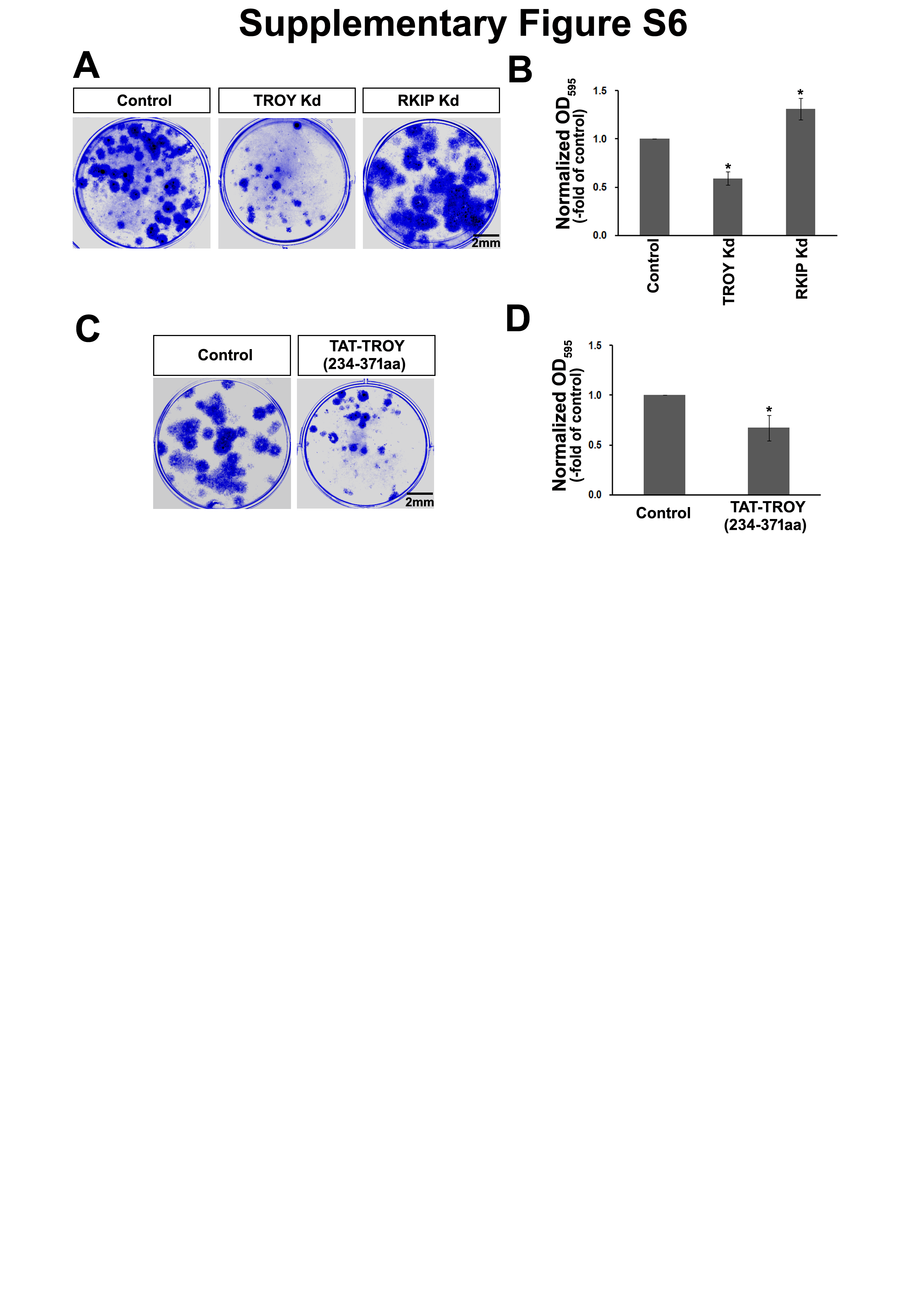

Supplement: Supplementary file 7 — Supplementary Figure S6 [file 41388_2018_503_MOESM7_ESM.tif]
